# Sorafenib and 2,3,5-triiodobenzoic acid-loaded imageable microspheres for transarterial embolization of a liver tumor

**DOI:** 10.1038/s41598-017-00709-4

**Published:** 2017-04-03

**Authors:** Jin Woo Choi, Ju-Hwan Park, Hye Rim Cho, Jin Wook Chung, Dae-Duk Kim, Hyo-Cheol Kim, Hyun-Jong Cho

**Affiliations:** 1 Department of Radiology, Seoul National University College of Medicine, Seoul National University Hospital, Seoul, 03080 Republic of Korea; 2 College of Pharmacy and Research Institute of Pharmaceutical Sciences, Seoul National University, Seoul, 08826 Republic of Korea; 3 College of Pharmacy, Kangwon National University, Chuncheon, Gangwon 24341 Republic of Korea

## Abstract

Sorafenib (SOF; an angiogenesis inhibitor) and 2,3,5-triiodobenzoic acid (TIBA; a contrast agent for computed tomography imaging)-loaded poly(lactic-co-glycolic acid) (PLGA) microspheres (MSs) were fabricated. Embolization, drug delivery, and tracing the distribution of MSs for liver cancer therapy were accomplished with the developed MSs after their intra-arterial (IA) administration. SOF/TIBA/PLGA MSs with 24.8–28.5 µm mean diameters were prepared, and the sustained release of SOF from MSs was observed. Lower systemic exposure (represented as the area under the curve [AUC]) and maximum drug concentration in plasma (C_max_) values of the SOF/TIBA/PLGA MSs group (IA administration, 1 mg/kg) in the results of the pharmacokinetic study imply alleviated unwanted systemic effects (*e.g*., hand and foot syndrome), compared to the SOF solution group (oral administration, 10 mg/kg). In a rat hepatoma model, the increase of microvessel density (MVD) following arterial embolization (*i.e*., reactive angiogenesis) was partially limited by SOF/TIBA/PLGA MSs. This resulted in the SOF/TIBA/PLGA MSs group (IA administration, single dosing, 1 mg/kg) showing a smaller tumor size increase and viable tumor portion compared to the TIBA/PLGA MSs group. These findings suggest that a developed SOF/TIBA/PLGA MS can be a promising therapeutic system for liver cancer using a transarterial embolization strategy.

## Introduction

Hepatocellular carcinoma (HCC) is the second leading-cause of cancer-related mortality in the world^1^. According to current guidelines, HCC can be curatively managed by liver transplantation, surgical resection, and local ablation^2^, but, unfortunately, only 30% of new HCC patients are eligible for the curative treatments^3^. The remaining patients undergo palliative treatments including transarterial chemoembolization (TACE), oral sorafenib (SOF), and supportive care. Besides, initially curable patients may receive palliative managements afterwards, when HCC recurs. In this context, TACE and SOF are two key players in current HCC management systems.

According to a recent global cohort study^4^, TACE is the most frequently used first-line treatment for HCC worldwide. In addition, TACE is the most common second-line treatment for patients who have already received a treatment other than TACE. This oncologic interventional method involves selective delivery of chemotherapeutic agents and embolic materials into the tumor-feeding arteries. The beneficial effect of TACE is mostly derived from arterial embolization^5, 6^, considering that HCC is typically hypervascular.

However, tumor hypoxia induced by arterial embolization causes vigorous angiogenesis following up-regulation of the hypoxia-inducible factor (HIF)-1α pathway; this consequently influences tumor recurrence and the survival of patients^7^. Therefore, studies of TACE in combination with the oral angiogenesis inhibitor are conducted for inoperable patients with HCC^8^. SOF, a multi-targeted tyrosine kinase inhibitor, is the most commonly used oral angiogenesis inhibitor; it is used not only as a monotherapy but also in combination with TACE. However, recent clinical trials of TACE with SOF imply that this combination can be a double-edged sword, showing worrisome toxicity profiles as well as promising clinical efficacy^9^.

Recently, various preparation methods of micro-sized carriers have been reported^10–17^. In our previous study^18^, doxorubicin (DOX)-loaded poly(lactic-co-glycolic acid) (PLGA) microspheres (MSs), fabricated by the solid-in-oil-in-water (S/O/W) method, were prepared; their application in the TACE of a liver tumor was evaluated. However, it is necessary to trace the *in vivo* fate of MSs after their administration to the hepatic artery to confirm the embolism and predict the therapeutic efficacies. An imaging agent that has conjugated or included polymeric MSs has been developed for the therapy of liver cancer^19, 20^. Notably, magnetic resonance imaging (MRI)-visible PLGA MSs, including SOF, have also been prepared and assessed *in vivo* ^21, 22^. In this study, PLGA MSs, including SOF and 2,3,5-triiodobenzoic acid (TIBA), were developed for transarterial embolization (TAE), locoregional delivery of SOF, and computed tomography (CT) imaging. They can maximize the embolic effect and angiogenesis inhibition, minimize the systemic toxicity of SOF, and monitor the lesion on follow-up CT imaging. PLGA has been widely used as a biocompatible and biodegradable polymer to prepare drug carriers^23, 24^. Considering its hydrophobic and degradable characteristics, it can be used for the fabrication of MSs and the sustained release of poorly water soluble drugs; as a result, biosafety can be accomplished^24^. It seems that TIBA may be one of the appropriate iodine derivatives for preparing particles with suitable physicochemical properties (*i.e*., dispersibility and sphericity), radiopacity, and biocompatibility^25–27^. Following fabrication, the particle size, biodegradability, drug release profiles, and CT imaging properties of MSs were characterized *in vitro*. After these initial *in vitro* characterizations, a rat model was employed to enable *in vivo* studies investigating pharmacokinetics, tumor responses, and CT imaging capabilities.

## Results

### Preparation and characterizations of SOF/TIBA/PLGA MSs

**Figure 1:**
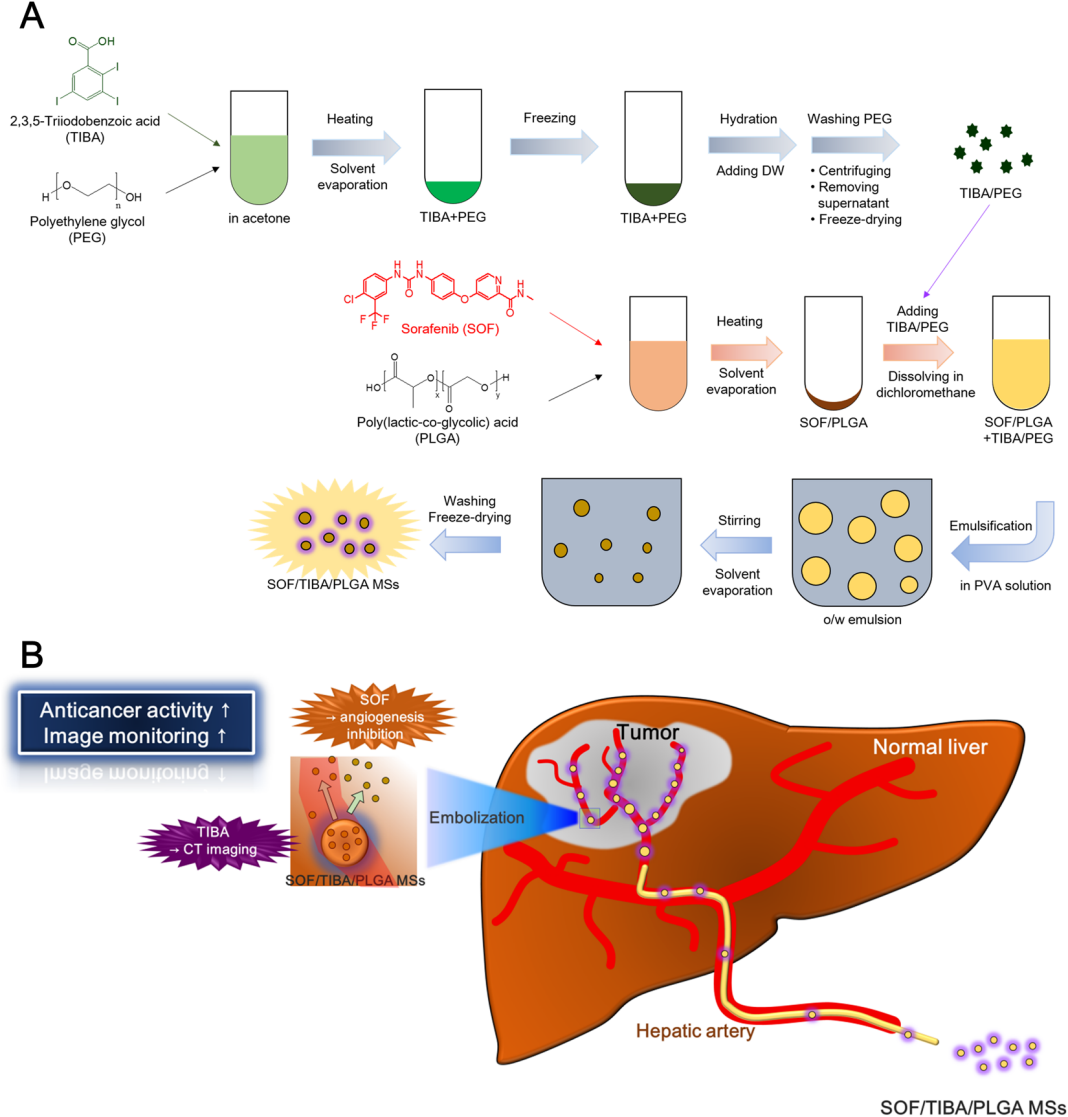
Schematic illustrations of developed MSs. (**A**) Fabrication process of SOF/TIBA/PLGA MSs is shown. (**B**) Therapeutic and imaging strategies of SOF/TIBA/PLGA MSs are presented. It is based on the illustration of the literature^28^ and permission was acquired from the publisher for its reuse.

SOF/TIBA/PLGA MSs were fabricated using a modified oil-in-water (O/W) emulsification method, as shown in Fig. 1A ^28^. When TIBA was added alone to the drug and polymer solution, it was easily released from MSs during the fabrication process, and a kind of precipitates were formed. Thus, to reduce the crystal size of TIBA and increase its content in MSs, TIBA dispersed in polyethylene glycol (PEG) was used instead of only TIBA. After heating (for solvent evaporation), freezing, and hydrating, the excess amount of PEG was washed off, and TIBA/PEG was obtained. In addition, due to the poor solubility of SOF in dichloromethane (DCM), SOF and PLGA were first dissolved in acetone, and an SOF/PLGA film was acquired after solvent evaporation. Prepared TIBA/PEG and SOF/PLGA were dissolved in DCM, and it was subsequently added to poly(vinyl alcohol) (PVA) solution. By emulsification and the solvent evaporation process, SOF/TIBA/PLGA MSs were finally prepared.

**Figure 2:**
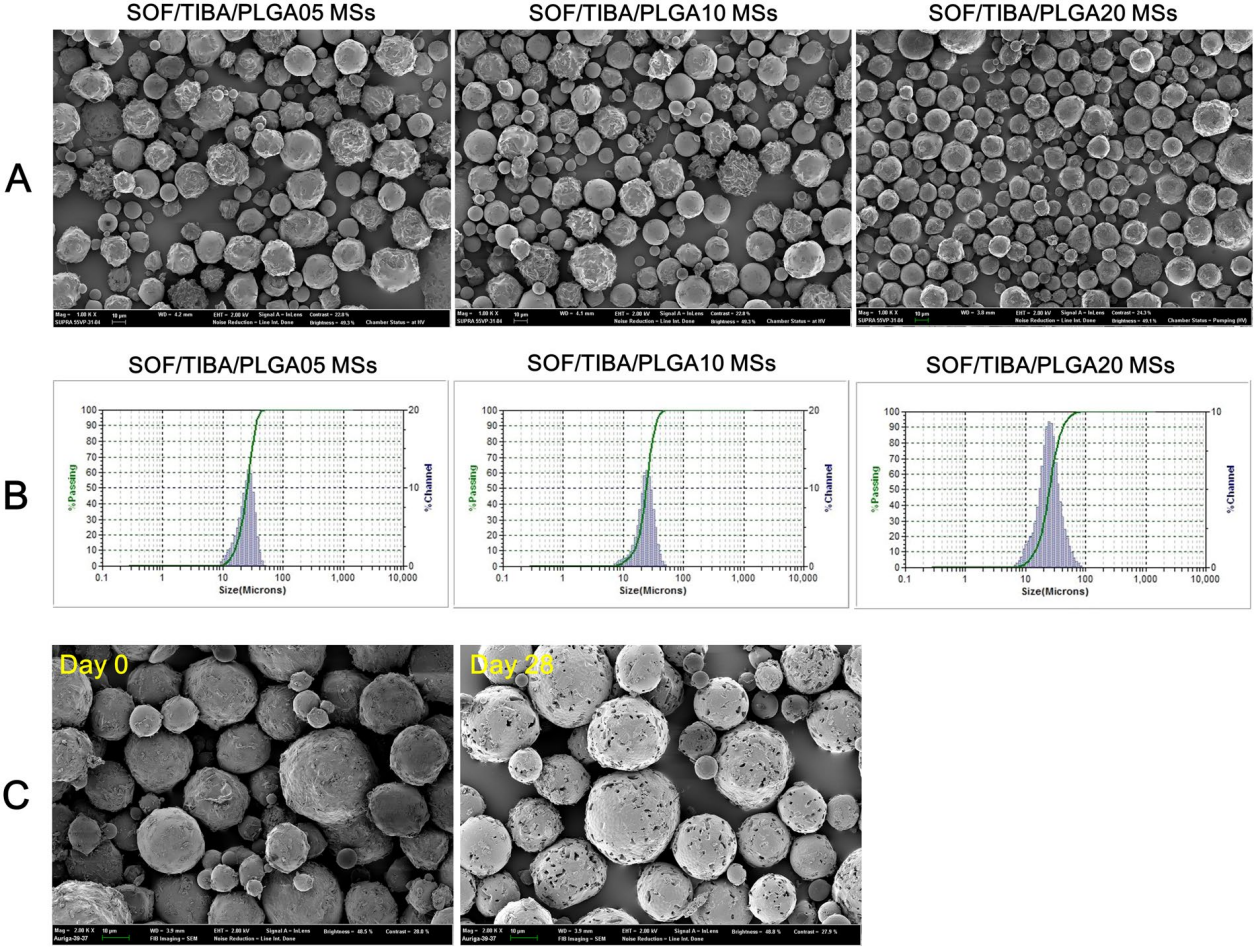
Physicochemical characterizations of developed SOF/TIBA/PLGA MSs. (**A**) FE-SEM images of SOF/TIBA/PLGA MSs are shown. The length of scale bar is 10 µm. (**B**) Size distribution charts of SOF/TIBA/PLGA MSs are presented. (**C**) FE-SEM images of SOF/TIBA/PLGA20 MSs at day 0 and day 28 after incubation in serum (50% FBS) are exhibited. The length of scale bar is 10 µm.

SOF/TIBA/PLGA MSs were administered to the hepatic artery for the embolization, therapy, and imaging of liver cancer (Fig. 1B). The mean diameters of developed MSs were 24.8–28.5 µm, and the average encapsulation efficiency values of SOF were 42.00–58.20% (Table 1). The mean contents (w/w) of SOF in SOF/TIBA/PLGA05 MSs, SOF/TIBA/PLGA10 MSs, and SOF/TIBA/PLGA20 MSs were 1.02%, 2.32%, and 5.11%, respectively. Also, the mean content (w/w) of iodine in SOF/TIBA/PLGA20 MSs was 23.15%. The mean diameter observed by the particle size analyzer was shown in the field emission-scanning electron microscope (FE-SEM) image, and a spherical shape was also presented (Fig. 2).

**Table 1:** Characterization of SOF/TIBA/PLGA MSs.

Composition	Mean diameter (μm)	SOF encapsulation efficiency (%)	SOF content (%)
SOF/TIBA/PLGA05 MSs (SOF:PLGA = 0.5:20)	25.1 ± 0.4	42.00 ± 1.01	1.02 ± 0.02
SOF/TIBA/PLGA10 MSs (SOF:PLGA = 1:20)	24.8 ± 0.3	48.70 ± 1.42	2.32 ± 0.07
SOF/TIBA/PLGA20 MSs (SOF:PLGA = 2:20)	28.5 ± 2.1	58.20 ± 0.79	5.11 ± 0.07

The ratio between SOF and PLGA is presented as the input amounts of materials.

Data are presented as mean ± standard deviation (SD) (*n* = 3).

$${\rm{{\rm E}}}\mathrm{ncapsulation}\,\mathrm{efficiency}( \% )=\frac{{\rm{actual}}\,{\rm{amount}}\,{\rm{of}}\,{\rm{SOF}}\,{\rm{in}}\,{\rm{MSs}}}{{\rm{input}}\,{\rm{amount}}\,{\rm{of}}\,{\rm{SOF}}\,{\rm{in}}\,{\rm{MSs}}}\times {\rm{100}}$$.

$${\rm{Drug}}\,\mathrm{content}( \% )=\frac{{\rm{actual}}\,{\rm{amount}}\,{\rm{of}}\,{\rm{SOF}}\,{\rm{in}}\,{\rm{MSs}}}{{\rm{amount}}\,{\rm{of}}\,\mathrm{SOF}-\mathrm{loaded}\,{\rm{MSs}}}\times {\rm{100}}$$.

### *In vitro* degradation of MSs

The biodegradability of SOF/TIBA/PLGA20 MSs in the bloodstream after intra-arterial (IA) administration was estimated by an *in vitro* stability test (Fig. 2C). The morphological shapes of SOF/TIBA/PLGA20 MSs after incubation in the serum for 28 days were observed by the FE-SEM image and compared with those of day 0 (pre). After 4 weeks of incubation in the serum, multi-pores were observed on the outer surface of MSs.

### *In vitro* SOF release

**Figure 3:**
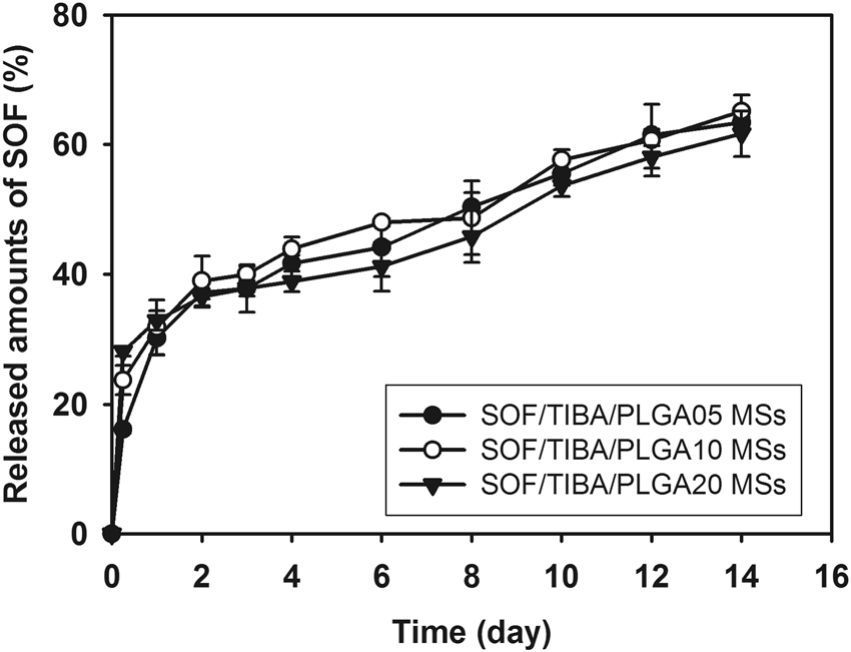
Drug release profile from SOF/TIBA/PLGA MSs (SOF/TIBA/PLGA05 MSs, SOF/TIBA/PLGA10 MSs, and SOF/TIBA/PLGA20 MSs) at pH 7.4. Each point indicates means ± SD (*n* = 3).

SOF release from developed MSs was assessed at pH 7.4 to predict the *in vivo* drug release pattern (Fig. 3). Released amounts of SOF from SOF/TIBA/PLGA05 MSs, SOF/TIBA/PLGA10 MSs, and SOF/TIBA/PLGA20 MSs on day 14 were 63.4 ± 0.9%, 65.1 ± 2.5%, and 61.7 ± 3.5%, respectively. The sustained drug release profiles (~14 days) from MSs were observed at pH 7.4 in all formulations.

### *In vitro* CT imaging of SOF/TIBA/PLGA MSs

**Figure 4:**
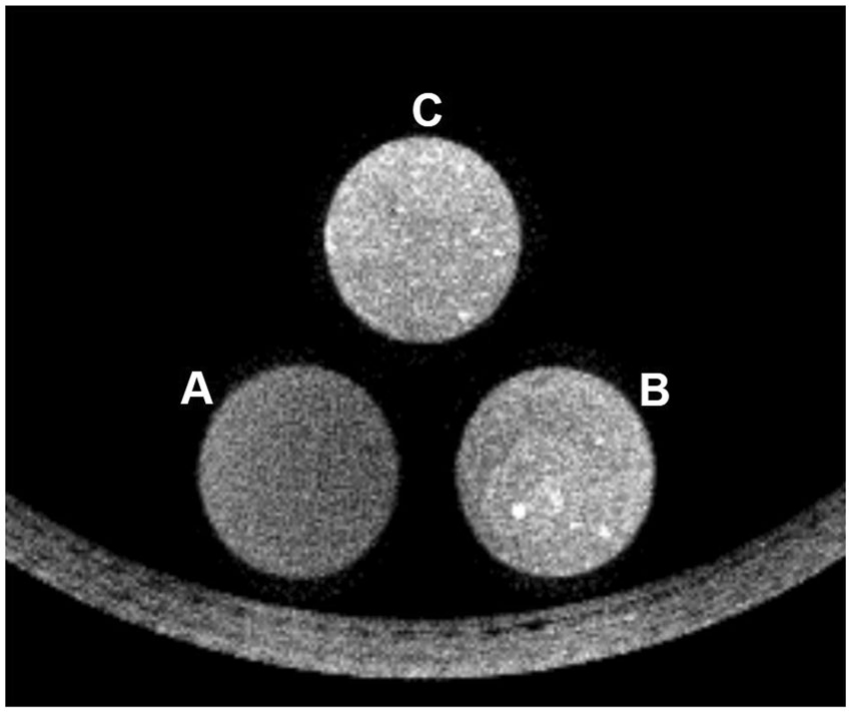
CT image of 2% agar phantom (**A**, control), 2% agar mixed with SOF/TIBA/PLGA20 MSs by manual pipetting (**B**), and 2% agar mixed with SOF/TIBA/PLGA20 MSs by sonication (**C**).

Two phantoms containing the SOF/TIBA/PLGA20 MSs demonstrated substantially higher attenuation compared with the 2% agar phantom (Fig. 4). The mean CT values were 130.7 HU, 293.7 HU, and 304.7 HU in the 2% agar, SOF/TIBA/PLGA20 MSs (manual pipetting), and SOF/TIBA/PLGA20 MSs (sonication), respectively. The SOF/TIBA/PLGA20 MSs (sonication) group (mean standard deviation [SD], 89.0) showed more homogeneous hyper-attenuation compared with the phantom (mean SD, 103.0) of the SOF/TIBA/PLGA20 MSs (manual pipetting) group. The signal-to-noise ratios (SNRs) (calculated by eq. (1)) were 1.7, 2.8, and 3.5 in the 2% agar, SOF/TIBA/PLGA20 MSs (manual pipetting), and SOF/TIBA/PLGA20 MSs (sonication), respectively.

### *In vivo* pharmacokinetics

**Figure 5:**
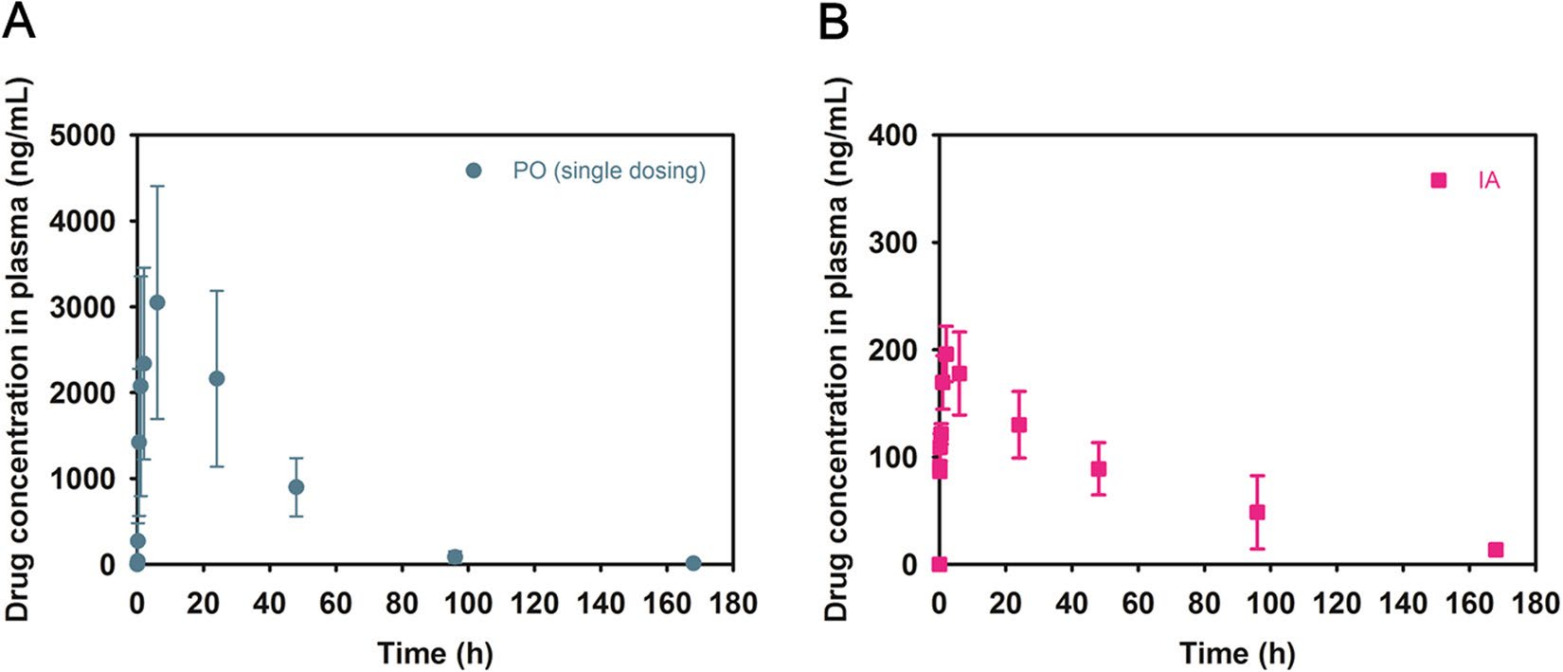
Pharmacokinetic profiles of SOF after oral (**A**) and IA (**B**) administration. SOF concentrations according to the time were presented. Doses of oral and IA administration were 10 and 1 mg/kg, respectively. Each point indicates mean ± SD (*n* ≥ 3).

SOF concentrations in plasma according to time were determined after oral administration of the SOF solution and IA administration of SOF/TIBA/PLGA20 MSs (Fig. 5 and Table 2). The total area under the plasma concentration-time curve from time zero to infinity (AUC) value of the oral administration group was significantly higher than that of the IA administration group (*p* < 0.05); however, there was no significant difference between the two groups after dose normalization. The maximum concentration (C_max_) value of the oral administration group was also much higher than that of the IA administration group (*p* < 0.05).

**Table 2:** Pharmacokinetic parameters of SOF after oral and IA administration in rats.

Parameter	SOF solution (oral)	SOF/TIBA/PLGA20 MSs (IA)
AUC (μg∙min/mL)	6804.0 ± 2650.1	717.6 ± 155.3*
AUC/D ((μg · min/mL)/(mg/kg))	680.0 ± 265.0	717.6 ± 155.2
C_max_ (ng/mL)	3048.7 ± 1356.5	196.2 ± 26.3*

*p < 0.05, compared with SOF solution (oral administration) group.

Doses of oral and IA administration were 10 and 1 mg/kg, respectively.

Data present as means ± SD (*n* ≥ 3).

### Drug distribution in the normal liver and liver tumor tissues

**Figure 6:**
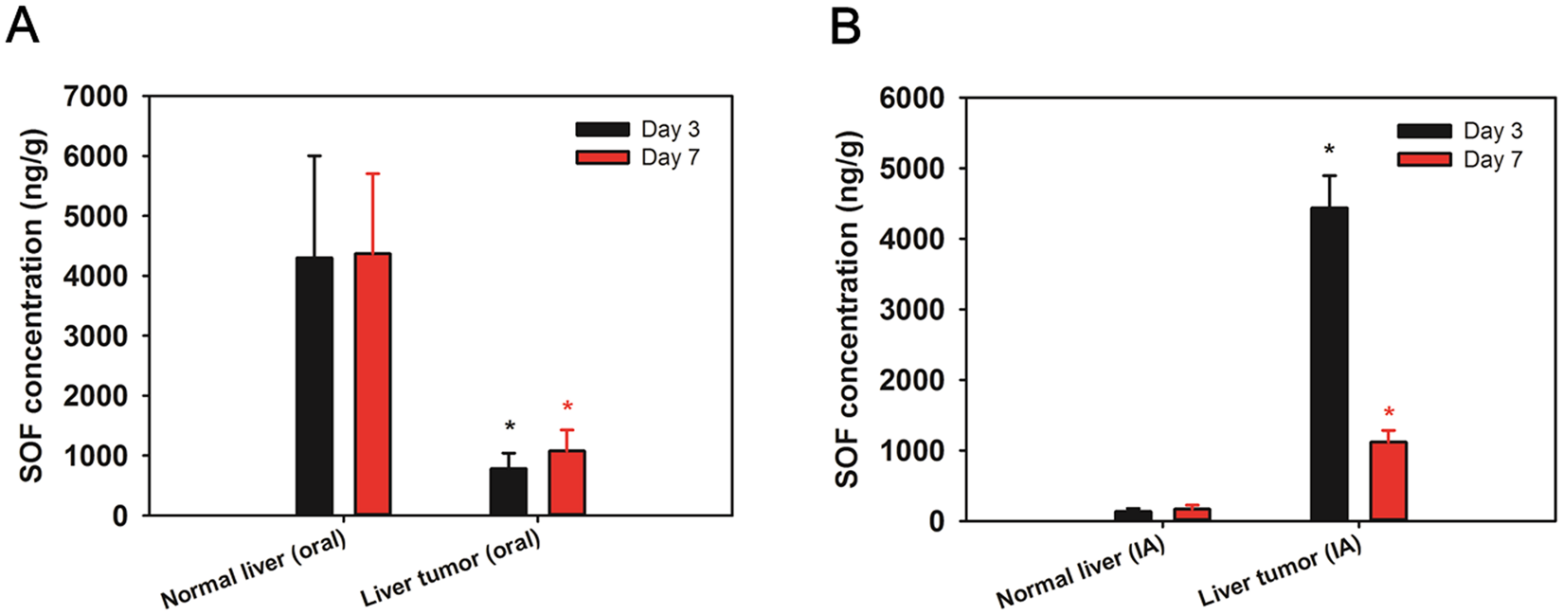
SOF concentrations in normal liver and liver tumor regions on day 3 and 7 after (**A**) oral administration of SOF solution (multiple dosing) and (**B**) IA administration of SOF/TIBA/PLGA20 MSs (single dosing). Doses of oral and IA administration were 10 and 1 mg/kg, respectively. Each point indicates means ± SD (*n* = 3).

After oral administration of the SOF solution (multiple dosing; 10 mg/kg/day dose) and IA administration of SOF/TIBA/PLGA20 MSs (single dosing; 1 mg/kg dose), drug concentrations in the normal liver and liver tumor regions were quantitatively determined (Fig. 6). In the oral administration group, the ratios of drug concentrations in the liver tumor compared to the normal liver on days 3 and 7 were 0.18 and 0.25, respectively. However, those ratios in the IA administration group on days 3 and 7 were 33.00 and 6.66, respectively. In spite of a lower dosing frequency and administration dose in the SOF/TIBA/PLGA20 MSs group (IA administration) compared with the SOF solution group (oral administration), the distribution ratio (liver tumor to normal liver) of SOF was significantly higher in the SOF/TIBA/PLGA20 MSs group than in the SOF solution group.

### Tumor response

**Figure 7:**
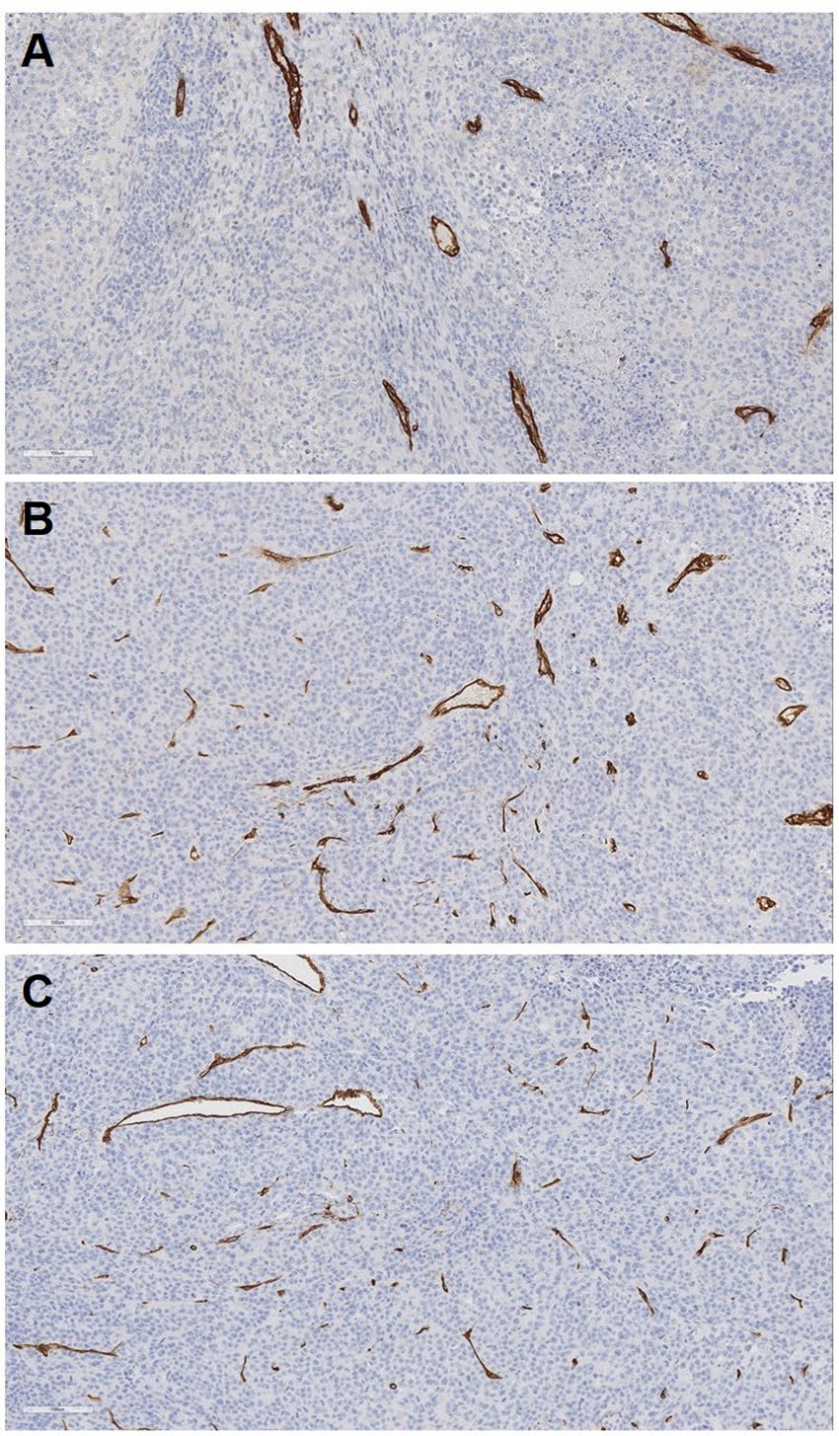
CD34 immunohistochemical staining of liver tissues treated by oral administration of SOF solution (**A**), IA administration of SOF/TIBA/PLGA20 MSs (**B**), and IA administration of TIBA/PLGA MSs (**C**), respectively.

Compared with the pre-treatment status, the tumor sizes increased by 115%, 111%, and 154% in the SOF solution (oral), SOF/TIBA/PLGA20 MSs (IA), and TIBA/PLGA MS (IA) groups, respectively. Viable tumor portions after the treatments were 29 ± 13%, 25 ± 11%, and 51 ± 13% in the SOF solution (oral), SOF/TIBA/PLGA20 MSs (IA), and TIBA/PLGA MS (IA) groups, respectively. The average values of microvessel density (MVD) were measured to be 14, 27, and 31 in the SOF solution (oral), SOF/TIBA/PLGA20 MSs (IA), and TIBA/PLGA MS (IA) groups, respectively (Fig. 7).

### *In vivo* toxicity

**Figure 8:**
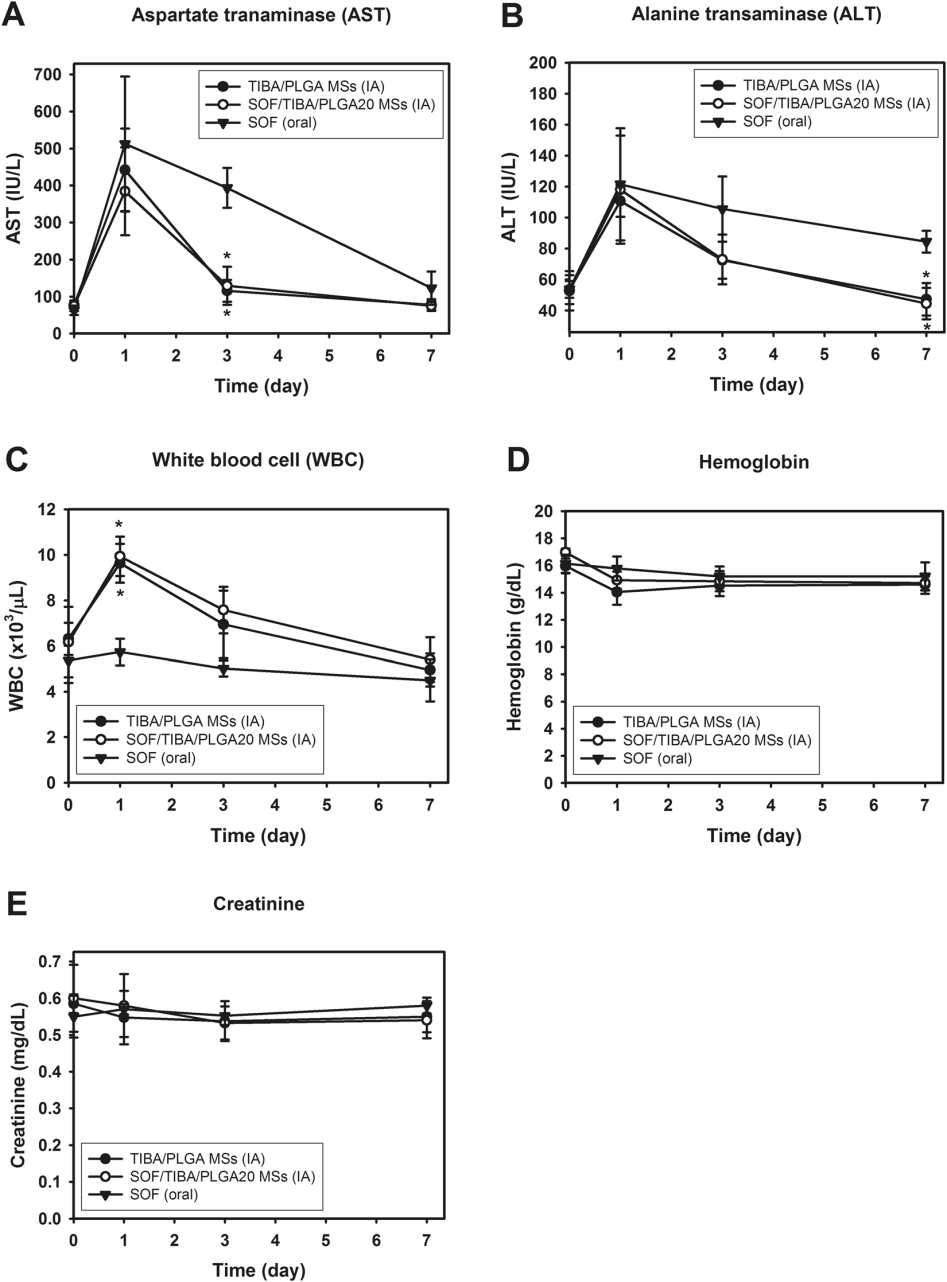
*In vivo* toxicity test after administration of SOF solution (oral), SOF/TIBA/PLGA20 MSs (IA), and TIBA/PLGA MSs (IA). The profiles of AST (**A**), ALT (**B**), WBC (**C**), hemoglobin (**D**), and creatinine (**E**) levels in the blood at day 0, 1, 3, and 7 are presented. Each point indicates means ± SD (*n* = 4). *p < 0.05, compared with SOF (oral) group.

Serum aspartate transaminase (AST) and alanine transaminase (ALT) levels were elevated at day 1 and then gradually decreased at day 3 and 7 in all groups, but the SOF solution (oral) group showed higher peaks at day 1 and slower normalization during the follow-up period compared to the SOF/TIBA/PLGA20 MS (IA) and TIBA/PLGA MS (IA) groups (Fig. 8). White blood cell (WBC) counts were substantially elevated at day 1 in the two IA groups, while relatively unchanged in the SOF solution (oral) group (Fig. 8). Hemoglobin and creatinine levels were consistent during the follow-up period in all groups.

### *In vitro* and *in vivo* CT monitoring of the SOF/TIBA/PLGA MSs

**Figure 9:**
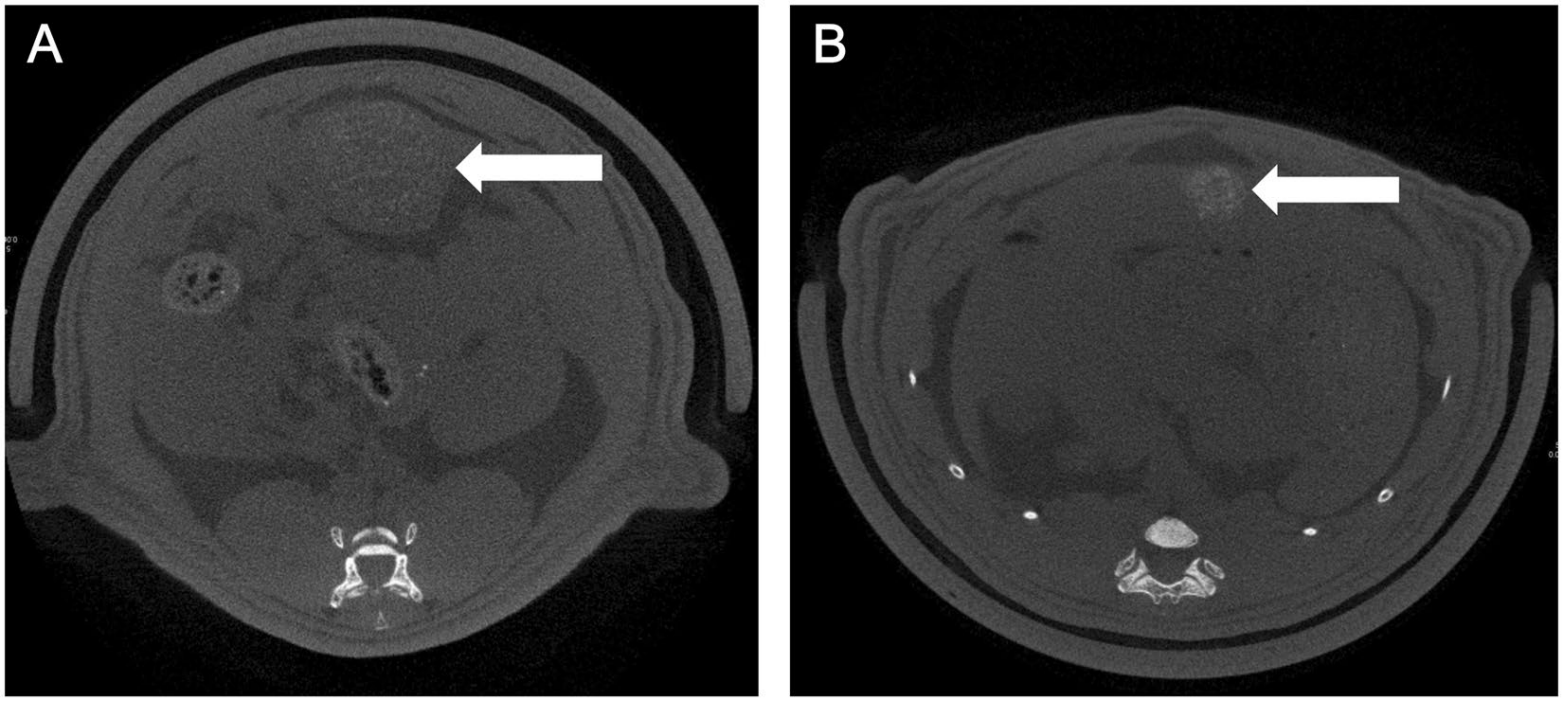
Hyper-attenuated spots appeared in the index tumor after IA administration of SOF/TIBA/PLGA20 MSs on CT images obtained on day 1 (**A**) and day 7 (**B**).

CT images on post-procedure 1 day clearly demonstrated hyper-attenuated spots, which had not been identified on the pre-procedural images in any of the five rats (Fig. 9). The spots persisted until day 7 in three out of the five rats (60%).

## Discussion

SOF has been used as an oral formulation but it can induce hand and foot syndrome, diarrhea, and hypertension^29^. These unwanted systemic effects may lead to the reduction or discontinuation of doses^30^. IA administration of SOF, compared with the oral administration of SOF, can reduce its systemic exposure and related side effects. For simultaneous embolization and chemotherapy of liver cancer after IA administration, various approaches have been tried, including emulsion composed of an anticancer agent and imaging agents, hydrogel, and MSs^18, 28, 31–33^. After the treatment, administered MSs need to be monitored by a noninvasive modality, such as CT, to trace the distribution of MSs and to predict the treatment response. In this investigation, TIBA as a CT imaging contrast agent was encapsulated with SOF into PLGA MSs, and the preparation method was optimized (Fig. 1). Although TIBA and SOF are hydrophobic, their other physicochemical properties (*i.e*., crystallinity, solubility in organic solvents) may hamper their direct physical loading onto the organic phase during the fabrication process of PLGA MSs. To prevent the precipitation of TIBA during the fabrication process of MSs and improve its encapsulation efficiency in MSs, PEG was used as a dispersing agent for TIBA. An excess amount of PEG was removed by the centrifugation step, and its dispersed form in PEG (TIBA/PEG) was obtained. Due to the poor solubility of SOF in DCM, an SOF/PLGA film was prepared by dissolving those in acetone and eliminating that solvent. TIBA/PEG and SOF/PLGA were added in DCM; subsequently, it was poured into the aqueous phase (PVA solution) to make O/W-type emulsion. By the emulsification-solvent evaporation step, hardened MSs were acquired.

The developed fabrication method also produced MSs (SOF/TIBA/PLGA05 MSs, SOF/TIBA/PLGA10 MSs, and SOF/TIBA/PLGA20 MSs) with an approximately 24.8–28.5 µm mean diameters (about 90% were ranged from 12 to 48 µm according to the volume distribution profile) (Table 1). For the embolization effect of MSs in rats, the particle size of MSs has to be matched to the internal diameters of the terminal arterioles (10–50 µm) and capillaries (8–10 µm) in the rat liver^34^. In addition, it was known that particles with a 40 µm mean diameter were necessary for the embolization of liver cancer in a rat model. Particles less than 40 µm may be distributed to the other organs and tissues, such as the lung and spleen^35^. As reported previously^28^, the particle size distribution of developed MSs may be suitable for the chemoembolization of liver cancer *via* the IA route (Fig. 2). A spherical shape and smooth surface, which are required for the blockade of internal vasculature, were also observed by FE-SEM imaging (Fig. 2). The SOF content was enough to meet the therapeutic dose for IA administration, and the iodine content was sufficient for tracing the *in vivo* fate of MSs by CT imaging.

The biodegradability of SOF/TIBA/PLGA20 MSs was evaluated in a serum (fetal bovine serum [FBS], 50%) solution, simulating the blood components. As shown in Fig. 2C, pores were formed after 4 weeks of incubation in the serum. PLGA can be degraded to lactic acid and glycolic acid *via* hydrolysis in the biological fluids. Biodegradation of PLGA MSs seemed to be closely related to sustained SOF release from MSs. Although blood flow and fluid dynamics may accelerate the degradation of PLGA MSs in the blood stream, *in vivo* biodegradability can be conveniently predicted by acquired *in vitro* stability data.

SOF release from fabricated MSs was assessed at pH 7.4 (Fig. 3). In general, sustained drug release has been regarded as one of the ideal properties of injection formulations. In the case of locoregional drug delivery for liver cancer therapy *via* the IA route, sustained drug release is crucial, as MSs have to block terminal arterioles and capillaries as well as provide a therapeutic agent for at least several days. In this study, drug release was maintained for 14 days and the drug release period in this study was extended further compared to the reported data with SOF-loaded PLGA MSs^21, 22^. By the optimized fabrication process (*i.e*., including complete solubilization of SOF and PLGA in acetone, film formation by solvent evaporation, dispersion of SOF/PLGA with TIBA/PEG in DCM, and subsequent emulsification), the drug may be entrapped efficiently and dispersed homogeneously in the MSs. It can lead to the sustained drug release profile, enhanced drug accumulation in the tumor region, and improved therapeutic efficacies. The biodegradation of PLGA MSs, observed in Fig. 2C, can be one of the mechanisms for the sustained drug release pattern. Also, there was no significant difference among the released amounts of SOF from SOF/TIBA/PLGA05 MSs, SOF/TIBA/PLGA10 MSs, and SOF/TIBA/PLGA20 MSs on day 14. At a fixed dose of SOF (1 mg/kg) for IA administration of MSs in animal studies, the required amounts of MSs may be increased as the weight ratio of SOF to PLGA decreased. The administration of excess amount of MSs can induce their reflux along the catheter during delivery and subsequent infarction in several organs. Considering the solubility of SOF in organic solvents (during the fabrication step of MSs) and reflux-related problems, SOF/TIBA/PLGA20 MSs seem to be appropriate for TACE in this study. Therefore, SOF/TIBA/PLGA20 MSs have been used in following animal studies.

The pharmacokinetic properties of SOF after the oral administration of the drug solution and IA administration of SOF/TIBA/PLGA20 MSs were assessed in normal rats (Fig. 5 and Table 2). Different doses for oral (10 mg/kg) and IA (1 mg/kg) administration were used while considering the clinical dose of SOF. The AUC value of the oral administration group was much higher than that of the IA administration group (*p* < 0.05); however, there was no significant difference in dose normalized AUC (AUC/D) values between the two groups. Higher systemic exposure, judged from the AUC value of the oral administration group compared to the IA administration group, may induce more unwanted systemic effects (*i.e*., hand and foot syndrome). The much higher C_max_ value of the oral administration group compared with the IA administration group may be also related to those side effects. Considering AUC and C_max_ values, the IA administration of MSs have more advantages in respect to reducing side effects in comparison with the oral administration group.

Drug distribution in the normal liver and liver tumor regions was evaluated in a liver cancer bearing rat model (Fig. 6). Despite low dosing frequency (single dosing) and the low dose (1 mg/kg) of the IA administration group compared to the oral administration group (multiple dosing, 10 mg/kg/day), the drug concentration ratio of the liver tumor compared to the normal liver was significantly higher in the IA administration group. This indicates that the selective drug accumulation in tumor tissues can be accomplished by IA administration. Sustained drug release from MSs and locoregional delivery to the liver can contribute to the selective drug uptake in tumor tissue after IA administration. It may ultimately reduce unwanted systemic toxicity to normal tissues and improve antitumor efficacy for liver cancer. Notably, the SOF/TIBA/PLGA20 MSs (IA, single dosing) group demonstrated milder liver toxicity compared to the SOF solution (oral, multiple dosing), which suggested that adverse reactions induced by multiple oral administration of SOF could be ameliorated by a single dose, IA delivery of the drug. In addition, the liver enzyme (AST and ALT) changes were almost identical in the two IA treatment groups. This result implies that the presence of SOF in MSs rarely contributes to the liver toxicity, and that initial liver enzyme elevation is mainly due to hepatic arterial embolization. The WBC increases in the two IA treatment groups seem to be substantially reduced in clinical practice. Instead of complex neck dissection for rats, in human, percutaneous puncture of the femoral artery is only required to deliver MSs to the liver.

The increase of MVD following arterial embolization (*i.e*., reactive angiogenesis) seems to be limited by SOF/TIBA/PLGA20 MSs (IA administration, single dosing, 1 mg/kg), which resulted in the SOF/TIBA/PLGA20 MSs group showing less tumor size increases and viable tumor portion compared to the TIBA/PLGA MSs group (IA administration, single dosing). This implies that SOF can elevate the antitumor efficacies in conjunction with the embolization property of MSs. Although the embolization of the tumor-feeding artery induces massive coagulation necrosis of the tumor, the activation of the HIF-1α pathway as an immediate response of hypoxia eventually causes local progression from the residual viable tumor^4^. Accordingly, clinicians have tried to combine the angiogenesis inhibitor (*e.g*., SOF) with TACE, but a recent randomized controlled trial revealed that the time to progression of HCC was not significantly improved by adding oral SOF to TACE^36^. This disappointing result may be due to the insufficient delivery of SOF to the tumor. By means of oral administration, the amount of SOF delivered to the tumor is relatively much lower than the total dose of intake. Furthermore, the blood flow to the tumor is very limited after TACE, suggesting that SOF in the blood may rarely reach the tumor region. In contrast, a much higher intratumoral concentration of SOF was presented in the SOF/TIBA/PLGA20 MSs group (compared to the SOF solution group) in this study, and it can explain the observed antitumor efficacies. Concordantly, post-embolization angiogenesis was less prominent in the SOF/TIBA/PLGA20 MSs group when compared to the TIBA/PLGA MSs group.

The SOF/TIBA/PLGA20 MSs were clearly identified on post-procedural CT images (Fig. 9). When the SOF/TIBA/PLGA20 MSs are delivered into the small arteries in a tumor, SOF may be distributed inversely proportional to the distance from the MSs. Therefore, noninvasive monitoring of the SOF/TIBA/PLGA20 MSs is needed for clinicians to predict an early tumor response after the treatment. For instance, if a 1-week follow-up CT scan showed focal washout of the SOF/TIBA/PLGA20 MSs in an index tumor, clinicians may provide immediate interventions, such as radiofrequency ablation or additional TACE for the MS-void area. This early intervention may prevent local recurrence generally identified on follow-up CT scans, and it may eventually improve patients’ survival rates.

## Conclusions

SOF- and TIBA-loaded PLGA MSs were fabricated by the modified O/W emulsification method. SOF/TIBA/PLGA MSs can be applied to the therapy of a liver tumor by the embolization, drug delivery, and monitoring *in vivo* fate of MSs by CT imaging. Developed SOF/TIBA/PLGA20 MSs with an approximately 29 µm mean diameter were appropriate for the embolization of the terminal arterioles and capillaries in the rat livers. The sustained release of SOF (for 14 days) may contribute to the enhanced antitumor efficacy after IA administration. According to the pharmacokinetic parameters (AUC and C_max_) in normal rats, IA administration of SOF/TIBA/PLGA20 MSs (single dosing, 1 mg/kg), compared with the oral administration of SOF (single dosing, 10 mg/kg), can reduce the unwanted systemic effects (*i.e*., hand and foot syndrome) of SOF. IA administration of SOF/TIBA/PLGA20 MSs (single dosing, 1 mg/kg) exhibited superior antitumor efficacies in a rat hepatoma model compared with that of TIBA/PLGA MSs (IA administration, single dosing). It showed more selective delivery of SOF to the tumor region compared with the oral administration group of SOF (multiple dosing, 10 mg/kg/day). Developed SOF/TIBA/PLGA MSs can be multifunctional systems for the therapy of liver cancer using a TAE method.

## Materials and Methods

### Materials

SOF was purchased from LC Laboratories (Woburn, MA, USA). TIBA was obtained from Tokyo Chemical Industry Co., Ltd. (Tokyo, Japan). PLGA (lactide:glycolide = 75:25, 66–107 kDa molecular weight), PEG (400 and 2,000 Da molecular weight), and PVA were acquired from Sigma-Aldrich Co. (St. Louis, MO, USA). FBS was purchased from Gibco Life Technologies, Inc. (Grand Island, NY, USA). All other reagents were of analytical grade.

### Preparation and characterization of SOF/TIBA/PLGA MSs

SOF and TIBA-loaded PLGA MSs were fabricated using a modified O/W method^28^. To improve the loading content of TIBA in MSs, TIBA dispersed in PEG (2 kDa) (TIBA/PEG) was prepared. TIBA (200 mg) and PEG (2 g) were dissolved in acetone (20 mL), and aliquots (1.8 mL) of TIBA/PEG were transferred to a tube. The organic solvent was eliminated by heating it to 80 °C under a gentle nitrogen gas stream. Melted TIBA/PEG was then rapidly moved into liquid nitrogen to be frozen. Distilled water (DW, 1 mL) was added to each tube, vortexed for 5 min, and centrifuged at 13,200 rpm for 3 min. After centrifugation, the supernatant was removed; this procedure was repeated three times. Remaining pellets were lyophilized for further use. SOF (0.5, 1, or 2 mg) and PLGA (20 mg) were solubilized in acetone (0.5 mL) due to the poor solubility of SOF in DCM. The acetone was then removed by heating it to 70 °C under a gentle nitrogen gas stream for 1 h. The prepared SOF/PLGA film and TIBA/PEG were dissolved in DCM (0.5 mL) and mixed. This organic phase was added to the PVA solution (2%, w/v; 5 mL), and the O/W emulsion was stirred for 5 min to remove the organic solvent. After centrifugation at 13,200 rpm for 3 min, the supernatant was removed, and the MS pellet was dispersed with DW. The dispersion of MSs was then freeze-dried and stored at −20 °C for further use.

The mean diameter of the SOF/TIBA/PLGA MSs was measured using a laser diffraction particle size analyzer (Microtrac S3500, Microtrac Inc., Montgomeryville, PA, USA) according to the manufacturer’s protocol. The morphology of MSs was also observed by a FE-SEM (SUPRA 55VP, Carl Zeiss, Oberkochen, Germany). MSs were coated with gold using sputter, and their morphological shapes were observed by the FE-SEM.

### Degradability of developed MSs

The *in vitro* degradability of SOF/TIBA/PLGA20 MSs was investigated in the serum. Prepared MSs (10 mg) were suspended in 50% (v/v) FBS mixed with phosphate-buffered saline (PBS) (0.5 mL), and it was incubated at 37 °C at 50 rpm for 4 weeks. To monitor the biodegradation of MSs by the FE-SEM, the dispersion of MSs was centrifuged at 13,200 rpm for 5 min, and MSs were collected for freeze-drying. Lyophilized samples were then observed by the FE-SEM (SUPRA 55VP, Carl Zeiss, Oberkochen, Germany) as described above.

### *In vitro* drug release

The amounts of SOF released from the MSs were measured at pH 7.4. SOF-loaded MSs containing 50 μg of the drug were suspended in PBS (pH 7.4), including 0.5% (w/v) Tween 80 (as a release medium), and incubated in a shaking bath (50 rpm) at 37 °C. Aliquots (200 μL) of the released media were collected at determined times (6, 24, 48, 72, 96, 144, 192, 240, 288, and 336 h), and an equivalent volume of fresh media was supplemented at each time. SOF concentration in the released media was determined by the high performance liquid chromatography (HPLC) method to understand the release profile of SOF. SOF was quantitatively determined using a Waters HPLC system (Waters Co., Milford, MA, USA) equipped with a separation module (Waters e2695; Waters Co.), a UV/Vis detector (Waters 2487; Waters Co.), and a column (reverse-phase, Fortis C18, 150 × 4.6 mm, 5 μm; Fortis Technologies Ltd., Cheshire, UK). The mobile phase was composed of DW containing 2% (w/v) triethylamine (pH 5.4 adjusted with phosphoric acid) and an acetonitrile mixture (30:70, v/v). The absorbance was detected at a 265 nm wavelength. The injection volume was 20 μL, and the flow rate was maintained at 1 mL/min. The lower limit of quantification (LLOQ) of the drug was 25 ng/mL, and the precision and accuracy of the used assay were within the acceptable range.

### *In vitro* CT imaging of SOF/TIBA/PLGA MSs

To investigate X-ray attenuation of SOF/TIBA/PLGA20 MSs, three different phantoms were made using 2 mL of 2% agar, 2 mL of 2% agar mixed with 10 mg of SOF/TIBA/PLGA20 MSs by manual pipetting, and 2 mL of 2% agar mixed with 10 mg of SOF/TIBA/PLGA20 MSs by sonication. Each group was added in the plastic tubes (1 cm in diameter) and scanned by a micro CT scanner (NFR Polaris-G90, NanoFocusRay, Jeonju, Korea). A radiologist selected three consecutive image slices that scanned the center of conical tubes, and the radiologist placed a round region-of-interest (ROI, 50 mm^2^) in each tube. The SNR was calculated by the following equation:

1 $${\rm{SNR}}=\,\frac{Mean\,of\,the\,CT\,values(HU)}{Standard\,deviation\,of\,the\,CT\,values(HU)}$$

### Animal procedures

The experimental protocols of animal studies were approved by the institutional Animal Care and Use Committee (Seoul National University College of Medicine, Seoul National University Hospital) and animal studies were conducted in accordance with those guidelines and regulations. Male Sprague-Dawley (SD) rats (Orient Bio, Sungnam, Korea) with an approximate body weight of 400 g were utilized, and they were reared in a light-controlled room at 22 ± 2 °C and at 55 ± 5% relative humidity. IA administration of MSs was conducted by an experienced interventional radiologist according to the previously reported protocol^28^. Under anesthesia with an intramuscular injection of a mixture of zolazepam (5 mg/kg, Zoletil^®^; Virbac, Carros, France) and xylazine (10 mg/kg, Rompun^®^; Bayer-Schering Pharma, Berlin, Germany), a 1.6-French microcatheter (Nano 1.6; Create Medic, Yokohama, Japan) was inserted into the carotid artery. With the guidance of X-ray fluoroscopy, the catheter was introduced to the proper hepatic artery, and the MSs suspended in the mixture of the normal saline and iodine contrast agent (Ultravist 370; Bayer Healthcare, Leverkusen, Germany) were infused *via* the microcatheter.

### *In vivo* pharmacokinetics

*In vivo* pharmacokinetics after oral administration of the SOF solution and IA administration of SOF/TIBA/PLGA20 MSs was assessed in normal rats (five rats for each group; single dosing). For oral administration of SOF solution, SOF was dissolved in an ethanol/PEG 400/DW mixture (20:50:30, v/v/v) at a concentration of 1.5 mg/mL. Considering the clinical dose of SOF and hepatic arterial blood volume of the rats, the doses of oral and IA administration were set to be 10 mg/kg/day and 1 mg/kg, respectively. Afterwards, aliquots of blood (200 μL) were collected from the tail vein at pre-determined times (5 min, 10 min, 30 min, 1 h, 2 h, 6 h, 24 h, 48 h, 96 h, and 168 h). Blood samples were centrifuged at 16,000 *g* at 4 °C for 3 min, and the supernatant was stored at −70 °C prior to the quantitative analysis of the drug.

SOF concentrations in plasma were quantitatively determined by a liquid chromatography-tandem mass (LC-MS/MS) system. Aliquots of plasma samples (50 µL) were blended with acetonitrile (450 µL) containing 0.5 µg/mL valsartan (internal standard [IS]) by vortex mixing for 5 min. After centrifuging at 13,200 rpm for 5 min, the aliquot (2 μL) of the supernatant was injected into the LC-MS/MS system equipped with an Agilent Technologies 1260 Infinity HPLC and Agilent Technologies 6430 Triple Quad LC/MS systems (Agilent Technologies, Wilmington, DE, USA). The electrospray ionization (ESI) source settings for the analysis of SOF and IS were optimized manually; gas temperature, gas flow, nebulizer pressure, and capillary voltage were 300 °C, 11 L/min, 15 psi, and 3,000 V, respectively. The fragmentation transition, fragmentor voltage, collision energy, and cell accelerator voltage for the analysis of SOF were *m/z* 465.2 to 270.3, 192 V, 24 eV, and 5 V, respectively. In addition, the fragmentation transition, fragmentor voltage, collision energy, and cell accelerator voltage for the analysis of IS were *m*/*z* 436.2 to 291.2, 98 V, 14 eV, and 5 V, respectively. Chromatographic separation of SOF and IS was performed using a Poroshell 120 EC-C18 column (50 × 4.6 mm, 2.7 µm; Agilent Technologies, Santa Clara, CA, USA). The mobile phase was composed of acetonitrile and a 0.2% (v/v) formic acid solution (80:20, v/v); the flow rate was 0.4 mL/min. Retention times of SOF and IS were 2.5 and 1.9 min, respectively. Acquisition and processing for the quantitative analysis of SOF and IS were performed with a MassHunter Workstation Software Quantitative Analysis (Version B.05.00; Agilent Technologies, Wilmington, DE, USA). Linearity was established in the range of 2.5–5,000 ng/mL SOF concentration. Precision and accuracy were shown within the acceptable ranges. The AUC, as a pharmacokinetic parameter, was calculated using WinNonlin software (ver. 3.1, Pharsight; Mountain View, CA, USA).

### *In vivo* antitumor efficacies and MRI

A rat hepatoma model was induced by inoculating 5 × 10^6^ N1-S1 cells (CRL-1604; ATCC, Manassas, VA, USA) in the liver of SD rats. To avoid spontaneous tumor regression^37^, cyclosporine A (20 mg/kg/day; Chong Kun Dang Pharmaceutical Corp., Seoul, Korea) had been subcutaneously injected for five days, from one day before the tumor implantation until four days after the tumor implantation^38^. Twelve days after the tumor induction, 30 tumor-bearing rats were scanned by a clinical magnetic resonance (MR) scanner, randomly divided into three groups, and treated by each protocol. Twice-daily oral administration of the SOF solution (multiple dosing, 10 mg/kg/day of SOF dose, *n* = 10), IA administration of the SOF/TIBA/PLGA20 MSs group (single dosing, 1 mg/kg of SOF dose, *n* = 10), and IA administration of the TIBA/PLGA MSs group (single dosing, *n* = 10) were performed. In the TIBA/PLGA MSs group, corresponding amounts of MSs to 1 mg/kg of SOF dose in SOF/TIBA/PLGA20 MSs were administered *via* the hepatic artery. The rats in each group were randomly divided into two subgroups, and each subgroup was sacrificed for further analyses on day 3 and day 7. The rats euthanized on day 7 underwent MR imaging just before their deaths. Based on the MR images, tumor sizes before and after the treatments were compared among the three groups. MR imaging was conducted with a T2-weighted imaging sequence (repetition time/echo time = 3,800/78 msec; bandwidth = 199 Hz/pixel; flip angle = 140°; slice thickness = 2 mm; field of view = 120 × 109 mm; matrix = 256 × 197). The normal livers and liver tumors were harvested after euthanasia. Each specimen was divided into two blocks; a half was submitted for histologic evaluation, and the other half was utilized to measure tissue distribution of SOF.

### *In vivo* tissue distribution of SOF

SOF concentrations in the normal liver and liver tumor regions of the SOF solution group (oral administration, multiple dosing, 10 mg/kg/day) and the SOF/TIBA/PLGA20 MSs group (IA administration, single dosing, 1 mg/kg) were determined. After drug administration *via* the oral route or the IA route, the liver was dissected on days 3 and 7. The normal liver and liver tumor tissues were separated to measure drug concentrations in each region. To determine the SOF concentrations in the specimens, each tissue was mixed with DW (1:4, w/w) and homogenized by ball milling. Drug concentrations in each tissue were measured using the LC-MS/MS method, which was described previously.

### Histologic analyses

Histologic analyses were conducted using the specimens obtained on day 7. Axial sections of the specimens were fixed in a 10% (v/v) buffered formaldehyde solution and were embedded in paraffin. Hematoxylin and eosin (H&E) and CD34 staining were conducted for each sample. Afterwards, a pathologist who was blinded to the treatment allocation visually assessed the percentage of viable tumor portion and MVD. To determine the MVD, the pathologist selected three hot spots per each rat with optical magnification (×200, 0.77 mm^2^), and the number of microvessels was counted. The mean value of three measurements per each rat was used for further analyses.

### *In vivo* toxicity

Twelve normal rats were randomly divided by three treatment groups: twice-daily oral administration of the SOF solution (multiple dosing, 10 mg/kg/day of SOF dose, *n* = 4), IA administration of the SOF/TIBA/PLGA20 MSs (single dosing, 1 mg/kg of SOF dose, *n* = 4), and IA administration of the TIBA/PLGA MSs (single dosing, *n* = 4) groups. Blood sampling was performed at day 0 (just before the treatments), 1, 3, and 7 in the tail veins, and AST, ALT, WBC counts, hemoglobin and creatinine were measured to evaluate systemic toxicity of each treatment.

### *In vivo* CT monitoring of SOF/TIBA/PLGA MSs

Among the rats in the SOF/TIBA/PLGA20 MSs group, the 7-day follow-up subgroup underwent unenhanced micro CT scans before the procedure on day 1 and day 7. The image sets were arranged in a random order, and they were addressed by the radiologist, who was unaware of the timing of the CT scans. The radiologist determined whether radiopaque materials appeared after the procedure and whether they were maintained during the follow-up period.

### Statistical analysis

Statistical analysis of the data was done using a two tailed *t*-test and analysis of variance (ANOVA). All experiments were repeated at least three times, and the data are shown as the means ± the SD.
